# Antithrombotic therapy in COVID-19

**DOI:** 10.12669/pjms.37.4.4607

**Published:** 2021

**Authors:** Sahrai Saeed, Øyvind Bleie

**Affiliations:** 1 Sahrai Saeed MD, PhD FESC Department of Heart Disease, Haukeland University Hospital, Bergen, Norway; 2 Øyvind Bleie MD, PhD, FESC Department of Heart Disease, Haukeland University Hospital, Bergen, Norway

The cardiovascular complications of Coronavirus disease 2019 (COVID-19) constitute acute cardiac injury (myopericarditis/myocarditis, myocardial infarction, stress cardiomyopathy, pericardial effusion/tamponade), cardiac arrhythmias and thromboembolic events including stroke, pulmonary embolism, cerebral venous thrombosis and venous thromboembolism (VTE.).^1^-^2^ Due to its systemic nature, COVID-19 affects vascular endothelium and leads to micro- and macrovascular thrombosis.^3^ The endothelial damage in COVID-19 may be caused by either direct invasion by the Coronavirus or through inflammatory-induced mechanisms. Inflammatory cytokines, endothelium activation and COVID-19 *per se* being a procoagulant state with increased platelet reactivity, all may lead to vascular thrombosis. In details, endothelial dysfunction, subintimal inflammation, hemorrhage, edema, dysregulation of vascular tone, as well as disseminated intravascular coagulopathy (DIC), sepsis induced coagulopathy (SIC), increased D-dimer/FDP (fibrin degradation factors) are all possible contributing factors to vascular thrombosis both on arterial and venous side.^1^-^4^ However, optimal antithrombotic treatment has so far not been established, and the results from ongoing randomized trials investigating low-molecular-weight heparin (LMWH) (enoxaparin), direct oral anticoagulants, aspirin and sulodexide for out-patients and hospitalized patients with COVID-19 are awaited.

In the recent edition of the *Pakistan Journal of Medical Science (Pak J Med Sci)* we read with great interest the article *“Aggressive thromboprophylaxis improves clinical process and decreases the need of Intensive Care Unit in Covid-19”* by Ugur and colleagues^5^ from Turkey. In their retrospective study, the authors included a total of 1216 hospitalized COVID-19 patients divided in two groups; Group-1 who were discharged directly to their homes and Group-2 who were transferred to an intensive care unit (ICU). The authors evaluated the effect of LMWH (enoxaparin) treatment on the rates of ICU transfer as well as readmissions. The likelihood for ICU stay was twice as high in patients who did not receive treatment with LMWH. Similarly, readmissions after discharge were higher in patients who did not receive enoxaparin in hospital. As known from other previous studies on COVID-19 patients, higher age, increased levels of D-Dimer and fibrinogen, and lower hemoglobin, platelets and lymphocytes values were associated with the risk of a need for ICU stay. The results by Ugur *et al*. are an important contribution to the literature. However, we would like to add some further insights to their report, as well highlight the importance of increased arterial stiffness in COVID-19 patients in the present expert commentary.

Our unpublished observations show that even in milder cases of COVID-19 recovered at home, patients may develop minor myocardial infarctions with typical symptoms, elevated cardiac troponins with or without ECG changes. A coronary angiography may show normal epicardial coronary arteries. Although global left ventricular ejection fraction is normal, a careful strain imaging on 2D speckle tracking echocardiography may show regional/segmental reduced global longitudinal strain reflecting ischemia. A cardiac MR in these cases is very useful and can reveal transmural late gadolinium enhancement (LGE) and microvascular obstruction by a thrombus in smaller transmural branches of the coronary arteries (*Mohamed Ali et al. 2021, unpublished observations*).

COVID-19-related myocardial injury may occur independent of the presence of cardiovascular risk factors. However, pre-existing target organ damage as reflected by increased arterial stiffness (arteriosclerosis) and atherosclerosis has a bidirectional cause-effect relationship with COVID-19 severity.^4^ Both target organ damage and COVID-19 disease severity share the same cardiovascular risk factors. Preliminary studies have shown that COVID-19 is associated with adverse functional and morphological changes in the arterial wall, although the long-term vascular consequences are yet to be explored.

Taken together, the results reported by *Ugur et al*. are important and directly relevant for the management of COVID-19 patients showing that through their anticoagulant and anti-inflammatory effects, treatment with LMWH/enoxaparin in hospitalized patients reduces the risk of transfer to ICU as well as readmissions. Furthermore, some patients with milder disease may initially not require hospitalization. They are, however, still in risk of developing silent or clinically evident minor myocardial infarctions during convalescent period. Hence, in the absence of dedicated guidelines on antithrombotic treatment in COVID-19 patients, a more liberal practice of using an aspirin may be beneficial, and should be considered in every individual patient.

## Authors Contribution:

Sahrai Saeed and Øyvind Bleie wrote, edited and approved the manuscript.
